# Timeliness of routine vaccination among 24–35 months children in Urban Gambia

**DOI:** 10.1371/journal.pone.0346048

**Published:** 2026-04-03

**Authors:** Bakalilu Kijera, Alieu Sowe, Mary Bobb, Sarja Jarjusey, Lamin F. Manjang, Salimina Sillah, Oghenebrume Wariri

**Affiliations:** 1 Expanded Program on Immunization, Ministry of Health, Banjul, The Gambia; 2 Directorate of Public Health Services, Ministry of Health, Banjul, The Gambia; 3 Epidemiology and Disease Control Program, Ministry of Health, Banjul, The Gambia; 4 PATH, Banjul, The Gambia; 5 Directorate of Research, Ministry of Health, Banjul, The Gambia; 6 National Health Insurance Scheme, Banjul, The Gambia; 7 Vaccines and Immunity Theme, MRC Unit The Gambia at London School of Hygiene and Tropical Medicine, Fajara, The Gambia; 8 Department of Infectious Disease Epidemiology, London School of Hygiene and Tropical Medicine, London, United Kingdom; CEA, FRANCE

## Abstract

**Introduction:**

Achieving the full benefits of immunization requires both high coverage and timely delivery of scheduled vaccines to provide maximum protection against specific infections. This study aimed to assess coverage and timeliness of routine childhood vaccinations among children in urban areas of The Gambia.

**Methods:**

A cross-sectional quantitative study was conducted among children in Western Region 1 (urban Gambia). A multi-stage cluster sampling method was employed, based on the WHO 30 X 7 cluster survey approach. Vaccination data were collected for children aged 24–35 months. Vaccinations were categorized as ‘early’, ‘timely’, or ‘delayed’ based on accepted vaccination windows as defined by the Gambia Expanded Program on Immunization (EPI). Data were analyzed using Kaplan–Meier curves to describe time to vaccination events.

**Results:**

Among the 355 children surveyed, 88.7% (315/355) had Infant Welfare Cards. Vaccines administered during the first year of life (0–11 months) had higher coverage than those given in the second year (12–23 months). The first dose of the pentavalent vaccine (Penta 1) had the highest timeliness at 75.4%, whereas the Oral Polio Vaccine booster (OPVb) recorded the least timeliness at 29.7%. The proportion of doses administered too early ranged from 4.1% to 11.5% for the first dose of measles-containing vaccine (MCV1) and OPVb, respectively. The highest proportion of delays was for the birth dose of the Hepatitis B vaccine (68.2%)—the proportion of children receiving the Pentavalent vaccine on time or early decreased with subsequent doses.

**Conclusion:**

Timeliness has improved compared with previous studies but remains below the target. However, additional efforts are required to drive further improvement, especially for the Hepatitis B vaccine and vaccines administered in the second year of life, to strengthen the control of relevant Vaccine Preventable Diseases (VPDs). Health workers should regularly conduct defaulter tracing to reach every child, to improve coverage and timeliness. Also, strengthen interpersonal communication on the importance of timely vaccination.

## Introduction

Immunization is the most cost-effective public health intervention for reducing morbidity and mortality from infectious diseases [1]. To maximize the benefits of scheduled vaccines, achieving high coverage and timely delivery is essential. Combining the two provides higher chances of optimal protection against infectious diseases [2]. While vaccination coverage is a key indicator of access, the timing of vaccine administration is equally important. Immunization programs can only be effective if children are vaccinated on time, before exposure to diseases, and not too early [3].

Delays in receiving the first vaccine dose in the primary series are a strong predictor of subsequent incomplete immunization [4]. Delayed vaccination leaves children unprotected and can lower herd immunity, contributing to outbreaks of Vaccine-Preventable Diseases (VPDs) [5]. A “timely dose” refers to a vaccine administered within the recommended age window, while “early dose” is one given either earlier than the minimum recommended age (i.e., early vaccination) or too soon after the previous dose in the series. A “delayed dose” is administered late, after the recommended timeframe for that vaccine, according to the national immunization schedule [6].

The Gambia introduced routine childhood immunization in May 1979 to reduce morbidity and mortality from VPDs. Currently, The Gambia Expanded Program on Immunization (EPI) provides 14 vaccines. These include one dose of Bacille Calmette Guérin (BCG) and Hepatitis B at birth; six doses of Oral Poliovirus Vaccine (OPV) at birth, and at 2, 3, 4, 9, and 18 months; three doses of Pentavalent (Penta) vaccine (combination of diphtheria, tetanus, pertussis, Hepatitis B, and Haemophilus influenzae type B) at 2, 3 and 4 months; three doses of the Pneumococcal vaccine (PCV) at 2, 3, and 4 months; two doses of Rotavirus (Rota) vaccine at 2 and 3 months; two doses of the Measles and Rubella (MR) vaccine at 9 and 18 months; one dose of the Yellow Fever vaccine at 9 months; Injectable Polio vaccine (IPV) at 4 and 9 months; the Meningococcal vaccine at 12 months; and the Diphtheria, Pertussis, Tetanus (DPT) vaccine, given one year after the last dose of Pentavalent vaccine [7]. All newborns are issued an Infant Welfare Card (IWC) to record their vaccination records.

Over the past decades, The Gambia routine childhood vaccination program has been highly successful. Consistently achieving coverage rates of at least 90%. However, many children continue to receive vaccines outside the recommended timeframes [8]. Vaccines are most effective when administered according to nationally recommended ages and dosing schedules. These are policies developed based on local epidemiology, disease burden, and immunological evidence. In The Gambia, the national immunization schedule is aligned with World Health Organization recommendations and tailored to maximize protection against vaccine-preventable diseases among children [9]. Timely vaccination provides direct protection to the vaccinated child. Therefore, it contributes to population-level protection by reducing the transmission of targeted pathogens and lowering the risk of infection among vulnerable populations. These include those with weakened immune systems or who cannot be vaccinated for medical reasons. [10]. For example, the timely administration of the birth dose of the Hepatitis B vaccine (HBV), within 24 hours, is critical in preventing mother-to-child transmission of the hepatitis B virus [11]. Long-term inflammation caused by the hepatitis B virus seriously damages the liver. It can lead to liver failure and cirrhosis, and can cause damage without causing symptoms [12]. The WHO strongly recommends the introduction of timely HBV in all countries’ routine vaccination programs [13,14].

In recent years, the Western Region 1 (WR1) of The Gambia, home to about 40% of the country’s population, has consistently recorded the lowest vaccination coverage rates, despite being the most urbanized and easily accessible area [15]. A clearer understanding of vaccination timeliness in the region is important. Studies have shown a “domino effect,” where untimely vaccinations (whether too early or delayed) in earlier doses increase the likelihood of subsequent delays, ultimately, or non-uptake of later doses [3,16,17]. This study, therefore, aimed to assess the coverage and timeliness of routine childhood vaccination among children in WR1. Improving vaccination coverage and timeliness in WR1 is crucial to the overall success of The Gambia’s immunization program. It is the key to achieving the ambitious goals of Immunization Agenda 2030 (IA2030).

## Method

### Study design

This was a cross-sectional quantitative study conducted in WR1 between March and July 2023. We recorded the immunization history of children from the IWC. The study population consisted of children aged 24–35 months born in 2020 and 2021. The WHO 30 X 7 cluster sampling method was adopted to select households.

### Study area

**Fig 1:**
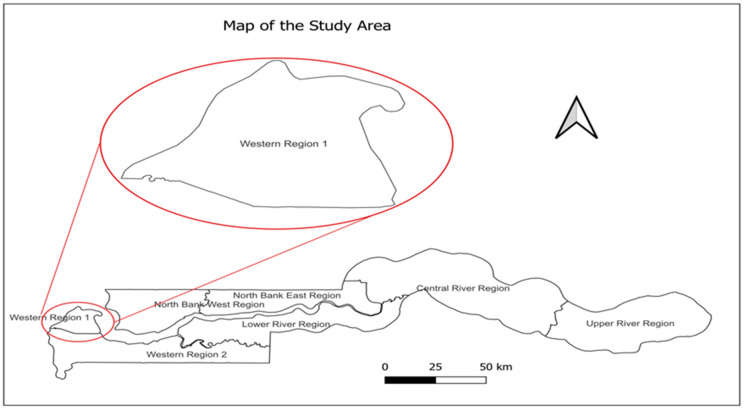
Map of the study area.

The study took place in WR1 of The Gambia (Fig 1), which has a total population of 1,021,836 people out of the country’s population of 2.4 million people [18]. Among this population, there are 31,432 children aged 12–23 months and 259,239 women of childbearing age [19]. WR1 is the most populous of The Gambia’s seven regions, comprising 40.4% of the national population. It comprises three districts—Banjul, Kanifing Municipality, and Kombo North. It has 34 health facilities (18 public and 16 private) offering immunization services. WR1 conducts 212 static clinic sessions and 19 outreach sessions monthly as of 2023. It is an urban region experiencing significant effects of rural-to-urban migration.

### Sample size determination

The MCV2 immunization coverage inThe Gambia was 71% according to The Gambia Demographic and Health Survey 2019-20 [15] and was used as a proportion in this study. All past demographic and health and multiple indicator cluster surveys have reported high vaccination card retention rates (more than 85%) [20]. With a confidence interval of 95% and a margin of error of 5%, the sample size was calculated as follows:


$$n=\frac{\overset{\text{2}}{\underset{\text{1}-\frac{\alpha }{\text{2}}}{Z}}P(\text{1}-P)\;}{{MOE}^{\text{2}}}$$

n = minimum required sample size

$\overset{\text{2}}{\underset{\text{1}-\frac{\alpha }{\text{2}}}{Z}}$= standard normal deviation 1.96 (CI 95% or 0.95)

P = 71% [15] for MCV2 coverage 2020

${MOE}^{\text{2}}$= the margin of error (5% or 0.05%)

Thus:


$$\text{n}=\frac{\;\text{1}.\text{9}{\text{6}}^{\text{2}}*\text{0}.\text{71}(\text{1}-\text{0}.\text{71})}{{\text{0}.\text{0}\text{5}}^{\text{2}}}$$

n = 317

To account for a 10% non-response rate, we added 32 participants, resulting in a total sample size of 349.

### Sampling method

We employed a multi-stage cluster sampling strategy, adopted from the WHO 30 X 7 cluster survey method, to ensure equal representation of each subpopulation [21]. The Gambia Bureau of Statistics randomly generated 30 clusters (enumeration areas) across the three districts in WR1. The distribution of clusters was as follows: 15 in Kombo North, 13 in Kanifing, and 2 in Banjul, allocated using probability proportionate to estimated size sampling. Systematic random sampling was used to select 12 households from each enumeration area, and one eligible child was randomly chosen from each household.

### Inclusion criteria

Children aged 24–35 months residing in WR1 were eligible for the study. The inclusion of Infant Welfare Card (IWC) was crucial as they provide accurate birth dates and vaccination dates. Those two are essential for calculating individuals’ ages at vaccination and assessing timeliness.

### Data collection method and processing

The research assistants collected the immunization records for children aged 24–35 months from 12^th^ April 2023 to28^th^ May 2023. The data entry was conducted using Android phones with customized questionnaires developed in KoboCollect. Data were systematically checked for completeness and plausibility. We applied built-in validation rules in the electronic data capture tool, including mandatory response fields for key variables, range checks for numeric values, and skip logic to prevent inapplicable responses. These validation measures minimized errors and prevented missing values. As a result, no missing data were observed in the final dataset. The final dataset was downloaded into Microsoft Excel and then exported to STATA version 17 for final data analysis.

### Classification of vaccine timeliness

We defined vaccination age in completed days as the interval between the date of birth and the date of vaccine administration. Scheduled ages followed The Gambia Expanded Programme on Immunization (EPI): BCG at birth; Penta 1 at 2 months; Penta 2 at 3 months; Penta 3 at 4 months; yellow fever and MR1 at 9 months; and MR2 at 18 months. We defined 1 month as 30 days for all age-at-vaccination calculations. This decision was made to align with the calendar-month language used in The Gambia EPI schedule and communication with caregivers and health workers. Accordingly, we operationalized scheduled ages as shown in Table 1. For each vaccine, we classified doses into three mutually exclusive categories, except for BCG and HBV, which were classified into two. Early: Administered before the scheduled age (e.g., < 60 days for Penta 1). Timely: Administered from the scheduled age through the subsequent 29 days (e.g., 60–89 days for Penta 1). Delayed: Administered at or after 30 days past the scheduled age (e.g., ≥ 90 days for Penta 1).

**Table 1:** The routine childhood immunization schedule in The Gambia as of 2023.

Vaccine	Vaccination window (Timely or age-appropriate vaccination)	Early Vaccination	Delayed Vaccination
HBV	≤24hrs	NA	>24hrs of life
BCG & OPV0	≤7days	NA	>7 days
OPV1, Penta1, PCV1 & Rota1	2 Months (60–89 days)	<60 days	>89 days
OPV2, Penta2, PCV2 & Rota2	3 Months (90–119 days)	<90 days	>119 days
OPV3, Penta3, PCV3 & IPV1	4 Months (120–149 days)	<120 days	>149 days
MCV1, OPV4, YF & IPV2	9 Months (270–299 days)	<270 days	>299 days
Men A	12 Months (360–389 days)	<360 days	>389 days
MCV2 & OPV Booster	18 Months (540–569 days)	<549 days	>569 days

The definition of “early” is programmatic—it reflects non-adherence to the recommended schedule. Early doses may still confer protective immunity and are recommended in specific clinical circumstances (e.g., measles vaccination for traveling infants) [22]. However, such doses do not satisfy routine schedule requirements and were therefore classified as non-timely in our primary analysis.

To assess the robustness of our findings to alternative month-length definitions, we repeated all timeliness classifications using the biologically standard definition of 1 month = 28 days for doses scheduled at ≤4 months of age; the differences observed were minimal.

### Data analysis

Vaccination coverage for vaccines administered during infancy and the Second Year of Life (2YL) was analysed using the information from the IWCs. A bar graph was developed to display vaccine coverage and timeliness. The timeliness of vaccinations was assessed using the Kaplan–Meier method, which is a survival analysis technique for time-to-event data. Kaplan–Meier curves were used descriptively to illustrate the timing of vaccine administration. No formal statistical comparisons between vaccines were performed. Vaccine timeliness was assessed only among children who received the respective vaccine dose; unvaccinated children were excluded from timeliness analyses.

### Ethical consideration

We obtained ethical approval from The Gambian Government/MRC Joint Ethics Committee, following an initial review of scientific content by the MRC Scientific Coordinating Committee (MRCG-SCC). The ethics committee includes government representatives, scientists, and heads of research departments. Caregivers were given written informed consent to review their child’s IWC.

## Results

### Routine vaccination coverage

**Fig 2:**
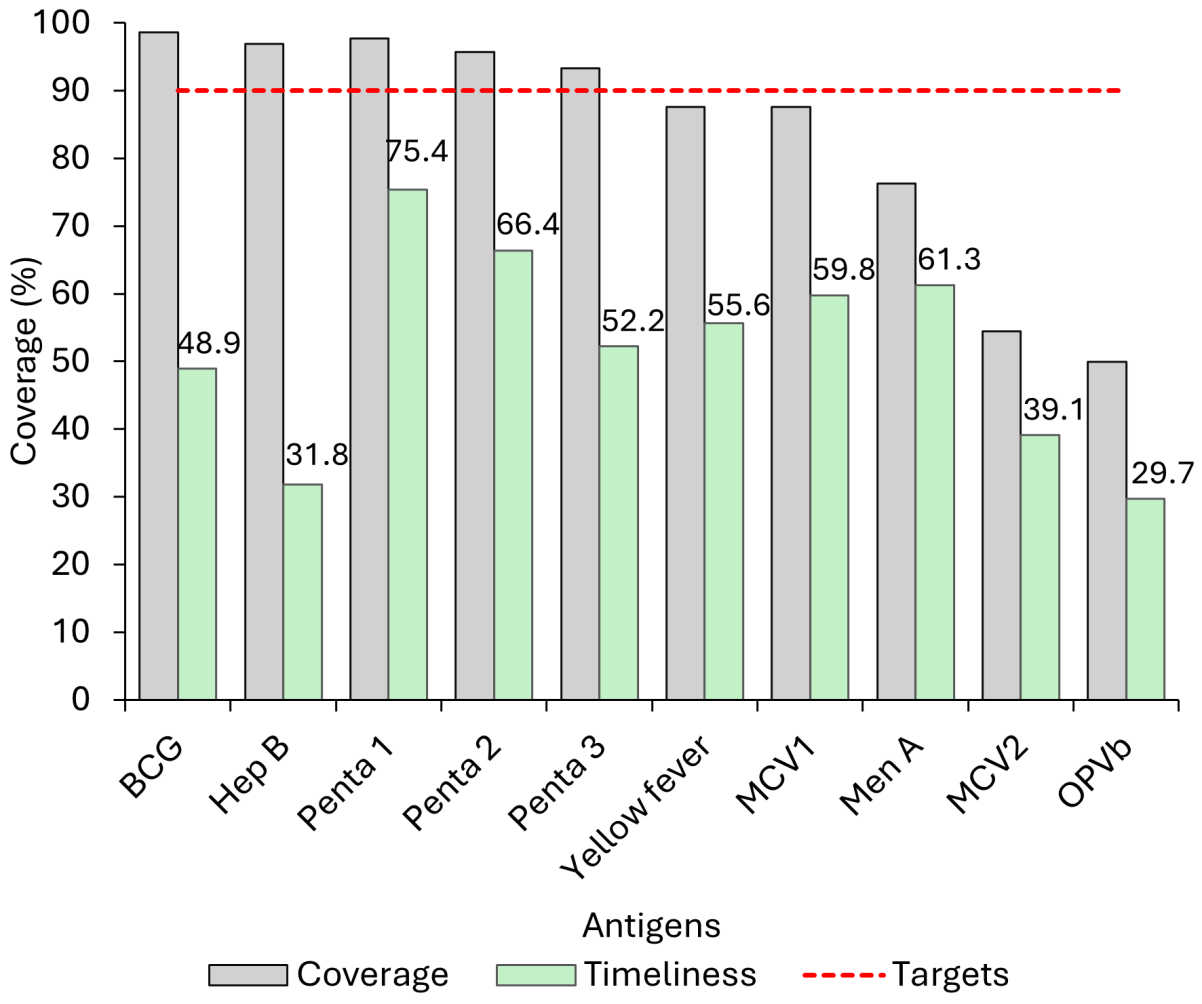
Vaccine coverage and timeliness among children 24–35 months, Western Region One, 2023.

Of the 355 caregivers interviewed, 315 had immunization cards for their children available for review, corresponding to an 89% card retention rate. The coverage of live birth doses (BCG & Hep B) and the Pentavalent vaccine series exceeded 90%. Coverage declined progressively for vaccines scheduled for older ages, indicating an increasing drop-out as children progressed through the immunization schedule. However, many of these vaccines are administered outside the age-appropriate vaccination schedule. Pentavalent dose 1 had the highest timeliness at 75.4%, and the Hepatitis B birth dose had 31.8% timely coverage (Fig 2).

### Early vaccination

In Fig 2, the timeliness of birth doses and the second year of life (12–23 months) was less than half. The proportion of early doses ranges from 4.14% for MCV1 to 11.49% for OPV Booster. Approximately12% received their OPVb vaccine early, while 9.84% received Pentavalent dose 1 earlier than recommended.

### Timely vaccination

**Fig 3:**
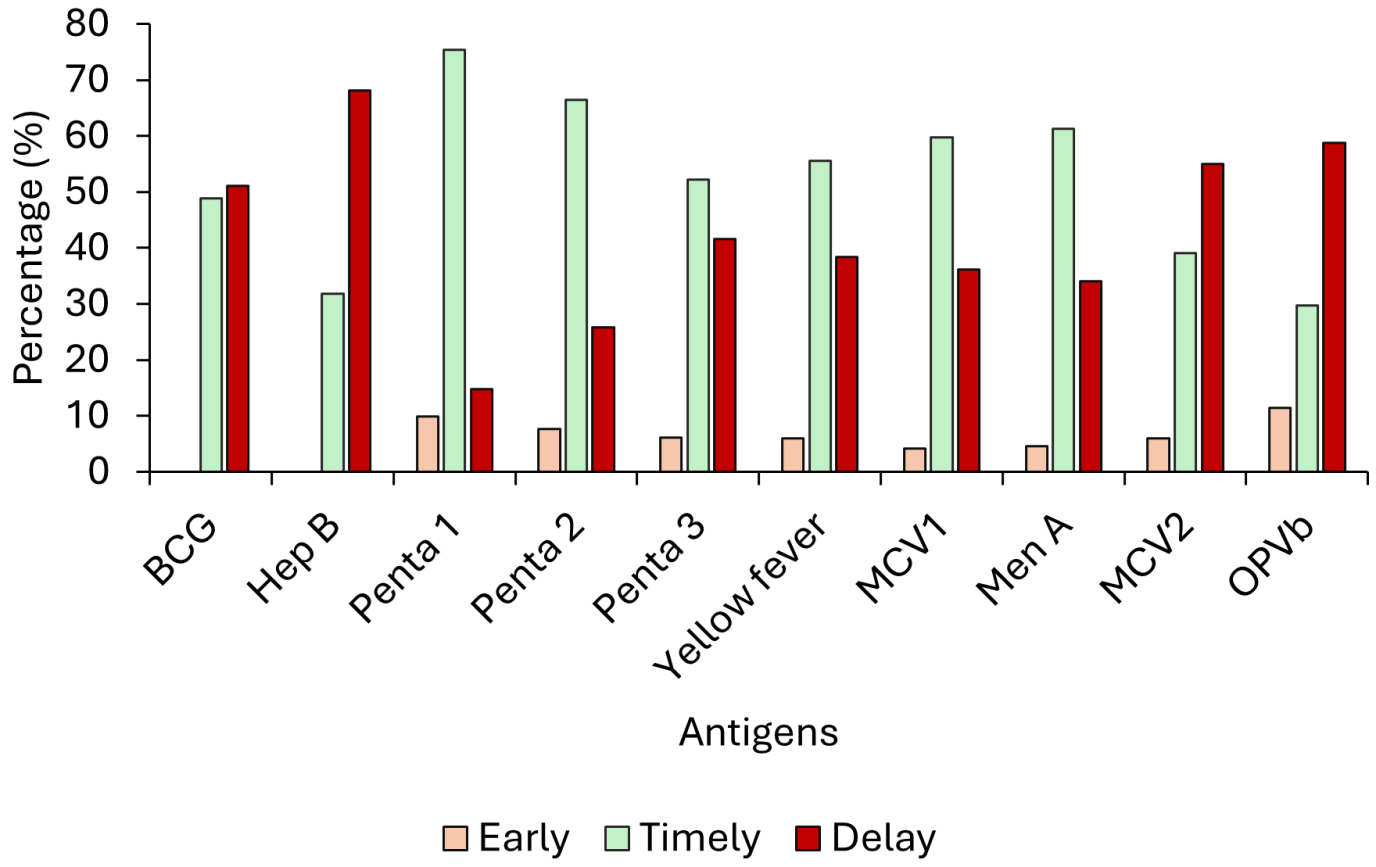
Timeliness of vaccination for specific vaccines among 24–35 months, Western Region One, 2023. Legend: Timeliness analyses were restricted to children who received the respective vaccines.

**Fig 4:**
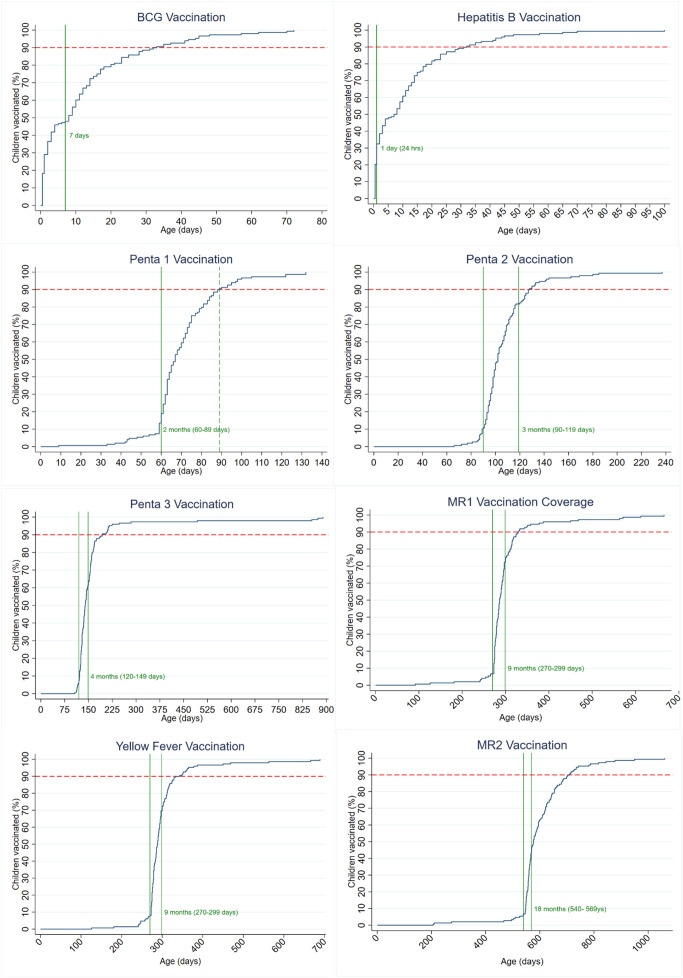
Cumulative routine vaccination coverage curve of children 24–35 months, Western Region One, 2023. **Legend:** Green vertical dash lines indicate the recommended national vaccination windows; BCG (0-7 days), Hep B (24 hrs of life), Penta 1 (60–89 days), Penta2 (90–119 days), Penta3 (120–149), MCV1 (270–299 days), Yellow Fever (270–299 days), and MR2 (540–569 days). The red dashed line shows the 2020 Global Vaccine Action Plan (GVAP) goal, at least 90% coverage.

Timeliness was above 50% for most vaccines, except for BCG (48.88%), Hep B (31.83%), MCV2 (39.1%) and OPVb (29.7%), as shown in Figs 3 and 4. The proportion of participants who received the Penta vaccine early or on time decreases by dose; 75.41% of infants received the first dose on time, but only 52.23% received the third dose on time, as illustrated in Figs 2–4.

### Delayed vaccination

Delayed vaccination timeliness was observed for all antigens but higher for birth doses and vaccines scheduled in the second year of life. The highest was Hep B vaccine (68.18%), followed by OPVb (58.78%). (See Figs 3 and 4).

## Discussion

The coverage of vaccines administered within the first four months of life exceeded 90%. Generally, vaccines given in the first year of life (0–11 months) had higher coverage compared to those administered in the second year of life (12–23 months). The Gambia is a high-performing country for BCG vaccination and has achieved the Global Vaccine Action Plan (GVAP) for Penta 3 vaccination [23]. Penta 1 recorded the highest timeliness of vaccine administration, while the OPV Booster recorded the least. However, delayed vaccination was the predominant form of untimely vaccination, occurring more frequently than early vaccination. Except for BCG and Hepatitis B, early administration was observed for other vaccines, indicating that some doses were given before the recommended age. For the multi-series vaccine (DPT-Hib-hep B), timeliness gradually reduced towards the third dose while the proportion of delayed administration increased.

High rates of untimely vaccination were observed in this study. This is similar to a study conducted in Ethiopia, where almost two-thirds of children were not vaccinated on time [24]. Although the timeliness of the birth dose vaccines (HBV and BCG) is less than half, there has been a significant increment in the timeliness of birth dose vaccines in The Gambia. In a large study assessing coverage and timeliness of the birth dose vaccines (HBV vaccine, BCG, and OPV) over 10 years (2004–2014) in The Gambia using the Farafenni Health and Demographic Surveillance System (FHDSS), the region recorded 1.1% of coverage at birth (day 0–1) [4]. In another study in The Gambia in 2019 and 2020, involving 41,720 children in the intervention group and 16,972 in the control group, the timeliness of the Hepatitis B birth dose for the intervention group were 16.0% and 23.6%, respectively, while the control group were 19.1% and 14.9%, respectively [25]. The timeliness of The Gambia Demographic and Health Survey (DHS), 2019–2020 was 15.0% [8]. In recent years, suboptimal immunization coverage has been observed in urban areas of The Gambia, primarily due to challenges inherent to urban immunization systems. This includes less cohesive social structures, challenges in defaulter tracing, and a high population-to-facility ratio in urban areas, which leads to long waiting times, among others [26]. Conversely, rural areas have reported higher coverage rates and may also record higher timeliness in vaccine administration [27]. Our study did not assess urban–rural differences; further research in rural Gambia could determine routine vaccine timeliness and identify best practices that could inform national strategies.

The Gambia Expanded Program on Immunization has implemented multiple interventions to improve the timeliness of birth-dose vaccination. All referral hospitals are equipped with standard cold-chain equipment, and immunization staff are deployed to those facilities to improve HBV timeliness. In addition, in some health facilities, Hepatitis B vaccination is a prerequisite for discharge; newborns must receive the HBV vaccine before their mothers can be discharged, in line with a policy directive of the Director of Health Services. Hep B vaccines had the highest proportion of delay. If children receive the Hepatitis B vaccine outside the recommended schedule or receive extra doses due to schedule overlap (e.g., a monovalent birth dose followed by hepatitis B antigens contained within a later pentavalent vaccine), this represents a vaccination program error; doses given too early than the recommended intervals may be invalid and require re-administration to complete the series appropriately [28].

The timeliness of Penta 1 (75.41%) was lower than that of a study in North-West Ethiopia (80.1%) [24]. In this study, we calculated Penta 1 timeliness as administration at ≥2 months (60 days), whereas some health workers administer it at ≥56 days, considering 28 days as one month. This discrepancy may explain the 9.84% earlier doses of Penta 1. We found that the timely coverage of later vaccine doses subsequently declines. For instance, the timely coverage of Penta 3 was lower than that of Penta 2 and Penta 1, and MCV2 was also lower than MCV1. Similar findings were found in Ethiopia and Nepal [24,29]. This could be due to mothers’ low awareness of immunization schedules. Adverse Events Following Immunization, such as abscesses at the injection site caused by the prior dose, may prevent mothers from taking their children to the next scheduled visits [30,31].

We also found that Yellow Fever had lower vaccination timeliness than MCV1. These vaccines are recommended at 9 months in The Gambia’s routine immunization schedule. The discrepancies could be because of a shortage of the Yellow Fever vaccine. It is important to note that any delay in receiving scheduled vaccines exposes children to infectious diseases. In this study, we found a high proportion of delayed vaccination among birth-dose vaccines and 2YL vaccines. The high proportion of delayed timeliness of MCV2 and OPV booster vaccines could be due to missed children who were vaccinated during the Periodic Intensification of Routine Vaccination in the communities.

This study provides an important update to the findings of the 2019–2020 Demographic and Health Surveys Program, which reported low timeliness of the Hepatitis B vaccine. Since that survey period, several intervention strategies have been introduced to improve HBV birth-dose timeliness. The present study offers more recent evidence from the largest region of The Gambia, which hosts the highest number of health facilities and major referral centres in the country. Furthermore, the data were collected directly from the community, providing a more representative assessment of vaccination timeliness at the population level. Despite these strengths, children within this age range were considered developmentally comparable. They were born across different calendar years. As a result, vaccination timeliness may have been influenced by temporal variations in immunization service delivery, vaccine availability, outreach activities, or programmatic interventions, which could not be fully assessed in this study. We defined 1 month as 30 days for all age calculations. While this deviates from the CDC-recommended standard of 28 days for intervals ≤3 months and could lead to potential misclassification. Although Kaplan–Meier curves suggested differences in vaccination timing across vaccines, no formal statistical tests were conducted to compare timeliness between vaccines with similar recommended ages. These findings should therefore be interpreted descriptively rather than inferentially.

## Conclusion

The immunization performance indicators are mostly centred on vaccine coverage and drop-outs. The delay in vaccination leads to a false assumption of protection against VPDs. This study indicated that vaccination timeliness had improved, but below the target. However, additional efforts are required to drive further improvement, especially for the Hepatitis B vaccine and vaccines administered in the second year of life. This will strengthen the control of relevant VPDs. This study revealed a substantial proportion of untimely vaccine administration, despite high vaccine coverage. Health workers should regularly conduct defaulter tracing to reach every child, improve coverage, and timeliness. Also, they should strengthen interpersonal communication on the importance of timely vaccination.

## Supporting information

S1 File Caregiver 2YL vaccination. (XLSX)

S2 File Inclusivity in global research questionnaire. (DOCX)
